# Highly Disturbed Populations of Seagrass Show Increased Resilience but Lower Genotypic Diversity

**DOI:** 10.3389/fpls.2018.00894

**Published:** 2018-06-29

**Authors:** Rod M. Connolly, Timothy M. Smith, Paul S. Maxwell, Andrew D. Olds, Peter I. Macreadie, Craig D. H. Sherman

**Affiliations:** ^1^Australian Rivers Institute – Coast and Estuaries, School of Environment and Science, Griffith University, Southport, QLD, Australia; ^2^Centre for Integrative Ecology, School of Life and Environmental Sciences, Deakin University, Geelong, VIC, Australia; ^3^School of Life and Environmental Sciences, University of Newcastle, Ourimbah, NSW, Australia; ^4^Healthy Land and Water, Brisbane, QLD, Australia; ^5^School of Science and Engineering, University of the Sunshine Coast, Maroochydore, QLD, Australia; ^6^Centre for Integrative Ecology, School of Life and Environmental Sciences, Deakin University, Geelong, VIC, Australia

**Keywords:** resilience, genotypic diversity, seagrass, *Zostera muelleri*, disturbance

## Abstract

The response of seagrass systems to a severe disturbance provides an opportunity to quantify the degree of resilience in different meadows, and subsequently to test whether there is a genetic basis to resilience. We used existing data on levels of long-standing disturbance from poor water quality, and the responses of seagrass (*Zostera muelleri*) after an extreme flood event in Moreton Bay, Queensland, Australia. Sites were grouped into high and low disturbance categories, in which seagrass showed high and low resilience, respectively, as determined by measuring rates of key feedback processes (nutrient removal, suppression of sediment resuspension, and algal grazing), and physiological and morphological traits. Theoretically, meadows with higher genotypic diversity would be expected to have greater resilience. However, because the more resilient meadows occur in areas historically exposed to high disturbance, the alternative is also possible, that selection will have resulted in a narrower, less diverse subset of genotypes than in less disturbed meadows. Levels of genotypic and genetic diversity (allelic richness) based on 11 microsatellite loci, were positively related (*R*^2^ = 0.58). Genotypic diversity was significantly lower at highly disturbed sites (*R* = 0.49) than at less disturbed sites (*R* = 0.61). Genotypic diversity also showed a negative trend with two morphological characteristics known to confer resilience on seagrass in Moreton Bay, leaf chlorophyll concentrations and seagrass biomass. Genetic diversity did not differ between disturbed and undisturbed sites. We postulate that the explanation for these results is historical selection for genotypes that confer protection against disturbance, reducing diversity in meadows that contemporarily show greater resilience.

## Introduction

The resilience of ecosystems to natural and anthropogenic disturbances is key to continued provision of services to humanity and support for biodiversity (Carpenter et al., 2012). The goal of enhancing resilience has thus been widely adopted in conservation management (Hughes et al., 2013), and this approach drives the imperative to better understand the factors underlying resilience. Resilience has two central elements: the capacity to resist change, and to recover after perturbations (Hodgson et al., 2015). While evolutionary biologists have long recognized the importance of genetic variation in supporting adaptation to changing environments (Fisher, 1930; Endler, 1986; Futuyma, 1986), the role of genetic diversity in promoting resistance and recovery has been studied only recently (Arnaud-Haond et al., 2010). Ecologists have begun to recognize the importance of genetic and genotypic diversity in shaping ecological characteristics, such as community diversity, structure and function (Vellend and Geber, 2005; Whitham et al., 2006; Hughes et al., 2008; Whitlock, 2014). Understanding the ecological effects of genetic and genotypic diversity is crucial for predicting how populations, communities and ecosystems will respond to environmental change, with several recent studies showing positive correlations between genetic diversity and a range of ecological processes. For example, higher levels of genotypic diversity, i.e., the number of distinct multilocus genotypes in a population or sample, has been shown to increase species richness of associated communities (Bangert et al., 2005; McArt et al., 2012; Whitlock, 2014), increase productivity (Crutsinger et al., 2006) and increase the recovery aspect of resilience (Reusch et al., 2005).

Several studies have used genotypic diversity when looking for positive effects of genetic diversity on ecosystem structure and functioning. This is because genotypes reflect genetically distinct individuals that may vary in ecologically important ways (i.e., they have distinct genomes and therefore vary across both neutral and adaptive loci). However, neutral genetic diversity is not always strongly correlated with adaptive diversity, and therefore nor with ecological processes, but may become associated indirectly through random genetic drift and migration acting at a location (Vellend and Geber, 2005; Whitlock, 2014).

Seagrass meadows provide a range of valuable ecosystem services, including carbon sequestration in underlying sediments (Macreadie et al., 2014a), shoreline stabilization, nutrient and sediment capture (Orth et al., 2006), habitat for economically important fish and crustaceans (Nagelkerken et al., 2015), and feeding grounds for turtles and dugongs (Heck et al., 2008). Unfortunately, seagrass habitat is also particularly vulnerable to human activities in the sea and in adjacent river catchments, because of rapid urbanization and industrialisation of coastlines around the world. Seagrasses have suffered very high rates of loss, degradation and fragmentation globally (Waycott et al., 2009; Short et al., 2011). While seagrass meadows often consist of just one or a few species, it is thought that genotypic diversity plays an equivalent role to species diversity in other ecosystems (Hughes and Stachowicz, 2011; Massa et al., 2013; Jahnke et al., 2015). Field and mesocosm studies have shown that higher levels of genotypic diversity are positively related to several ecological aspects, including: resistance (Hughes and Stachowicz, 2004; Massa et al., 2013; Evans et al., 2017), recovery (Hughes and Stachowicz, 2004, 2011), productivity and faunal abundance (Reusch et al., 2005), restoration success (Reynolds et al., 2012, 2013), mitigation of the effects of grazing (Hughes et al., 2010), and the ability to cope with the effects of climate change (Reusch et al., 2005; Ehlers et al., 2008). A meta-analysis of studies on *Posidonia oceanica* showed that genetic diversity (allelic diversity, genotypic richness, and observed heterozygosity) was weakly, but significantly, associated with the extents of several disturbance types, namely shipping, pollution, and cumulative impact (Jahnke et al., 2015). A broader meta-analysis across multiple species also found an overall positive relationship between genetic diversity and resilience (Salo and Gustafsson, 2016). Given the ecological and evolutionary importance of genetic variation to resilience and a wide range of ecosystem services, it is crucial to understand how levels of genetic and genotypic diversity determine the response of natural populations to disturbance (Unsworth et al., 2015; Connolly et al., 2018).

The seagrass of Moreton Bay, southeast Queensland, Australia, provides a useful setting for testing the effects of disturbance and the genetic basis of ecosystem resilience. Seagrass meadows in Moreton Bay have been exposed to widely divergent water quality since the forested landscape was altered by agricultural and urban development from the time of European settlement (Maxwell et al., 2015). The changing land-use in river catchments on the western side of the bay has resulted in long-standing water quality issues. Water clarity is reduced seasonally by high concentrations of suspended sediments and the proliferation of algae resulting from high nutrient loads (Leigh et al., 2013). Meadows in the western bay adjacent to the city of Brisbane have been greatly affected, and now cover only a fraction of their former distribution (Leigh et al., 2013). For the seagrass of Moreton Bay, we have existing estimates of resilience based on detailed measurements of environmental impact, ecological processes and biomass at 12 meadows of the dominant species, *Zostera muelleri*, over the 12 months following the flood (Maxwell et al., 2014). We also understand the links between resilience capacity and key morphometric measures of seagrass in the bay (Maxwell, 2014). Our aim was to test whether meadows exposed to long-standing differences in disturbance levels, and showing different capacities for resilience, differed in levels of genotypic and/or genetic diversity.

The severity of the long-standing disturbance to seagrass provides another advantage to the use of Moreton Bay seagrasses as a model system. Previous studies have suggested that the relationship between genotypic diversity and resilience is likely to be non-linear and only evident in the most extreme cases of disturbance (Arnaud-Haond et al., 2010). This suggests the occurrence of a threshold below which the relationship between genotypic diversity and resilience breaks down (Arnaud-Haond et al., 2010). Given the loss and fragmentation of meadows in the western bay, the environmental impacts can be considered extreme and therefore provide a robust test of the genetic basis of resilience.

## Materials and Methods

### Study Rationale and Design

**FIGURE 1:**
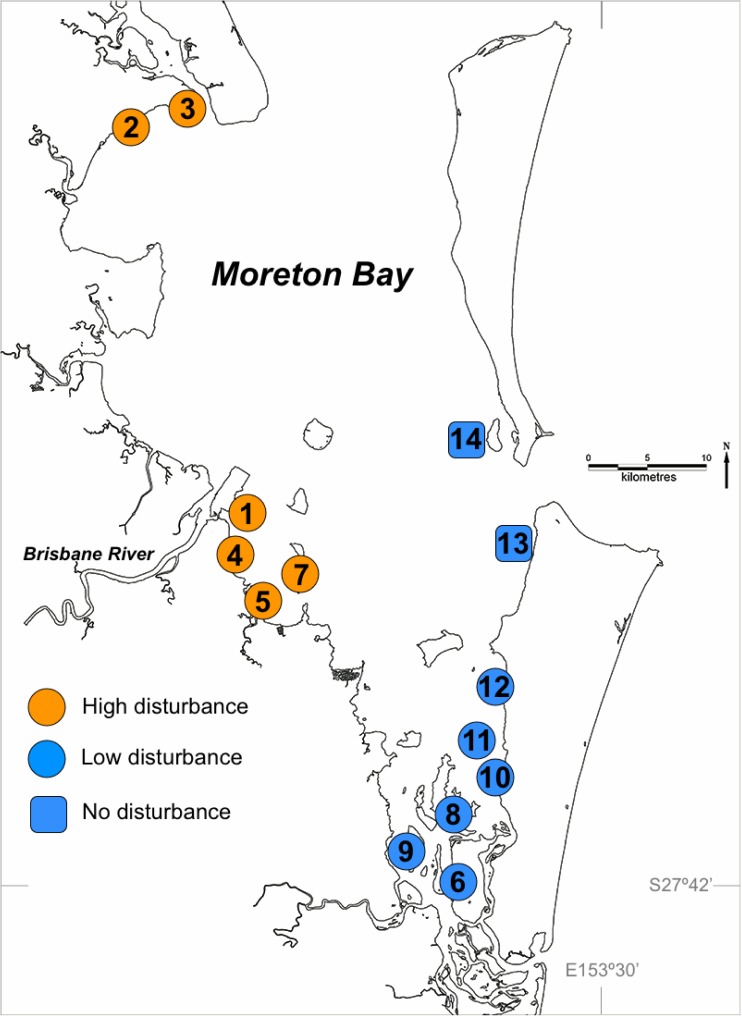
Seagrass sampling sites in Moreton Bay, Queensland, Australia, showing high and low disturbance categories. The two ‘No Disturbance’ sites (numbers 13 and 14) are treated as Low Disturbance throughout the paper, but for these two sites there were no resilience measures. Numbering is consistent with that in Maxwell et al., 2014.

This study examines levels of genotypic and genetic variability within and among *Z. muelleri* meadows in Moreton Bay (**Figure 1**) for which the disturbance regime and measures of seagrass resilience were previously established by Maxwell et al. (2014). Maxwell et al. (2014) used the response of seagrass systems to extreme flooding in Moreton Bay as an opportunity to test the interaction between the magnitude of historical disturbances, rates of ecological processes involved in critical feedback loops, measures of physiological and morphological changes, and changes in seagrass biomass. The 12 sampled meadows are grouped into two disturbance categories (see **Figure 1** for map and site numbering):

(1) high disturbance sites had poor and fluctuating water quality during summer months (wet season) over the previous 10 years due to riverine influence, quantified as high turbidity and nutrient concentrations and low salinity (*n* = 6 meadows); and
(2) low disturbance sites had historically better water quality during summer months with less riverine influence (*n* = 6 meadows).

**FIGURE 2:**
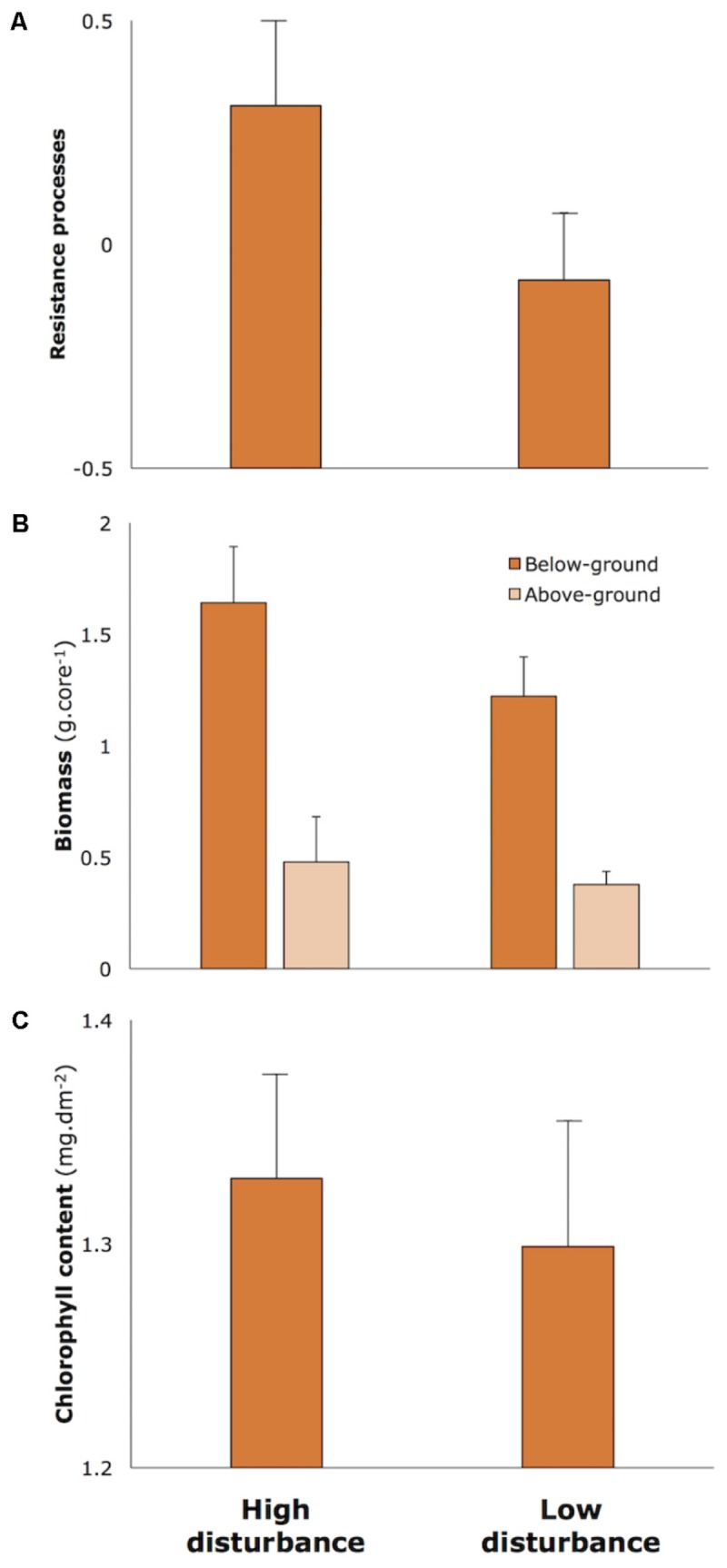
Measures of resilience and morphological characteristics of seagrass in High and Low Disturbance seagrass meadows in Moreton Bay (*n* = 6 in each category). **(A)** Resilience processes measure is a resistance score (range –1.0 to +1.0) along the main axis in multivariate space based on a combination of rates of three feedback processes (sediment suppression, nutrient removal, algal grazing) and changes in biomass, as explained in Appendix 1. **(B)** Biomass measured above and below-ground. **(C)** Chlorophyll a concentrations in leaves. Data redrawn from Maxwell et al., 2014.

Applying the measure of ecological resilience conceptualized in Maxwell et al. (2016), resilience for each meadow was quantified using a multivariate assessment of the rates of three key processes (sediment suppression, nutrient removal, algal grazing) and seagrass biomass through time. This process measure is detailed in Appendix 1. This multivariate measure clearly showed that resilience was higher in high-disturbance meadows than in low-disturbance meadows (**Figure 2A**, details in Table Appendix 1). Several morphological characteristics of seagrass are putatively related to resilience (Maxwell et al., 2015), and comparisons of characteristics between high and low disturbance meadows in Moreton Bay showed that high-disturbance, high-resilience meadows had on average higher above and below-ground biomass (**Figure 2B**) and higher concentrations of chlorophyll pigments (**Figure 2C**).

### Sample Collection for Genetic Analysis

Samples for genetic analysis were collected from a total of 14 sites (meadows) in Moreton Bay (**Figure 1**). Twelve sites are the same as those shown in **Figure 1** of (Maxwell et al., 2014), grouped as 6 high disturbance and 6 low disturbance sites, for which all seagrass variables and resilience measures are also available. Two additional sites were sampled for genetics, in an area of Moreton Bay that receives no riverine disturbance. Because these two sites received no flood impact in 2011 the full suite of process and morphological variables were not measured by Maxwell et al. (2014) since no estimate of resilience can be made where there is no disturbance at all. At each of the 14 sites, 15 samples were haphazardly collected from each of three, 10 × 10 m quadrats separated by approximately 100 m, for a total of 45 samples per site. Each sample consisted of a single shoot and a small section of rhizome. Samples were stored frozen prior to genetic analysis. The genetic samples were collected in October 2013, not long after the year of major flooding. However, the timing was not particularly important for our hypotheses, given our intention to test the effects of selection forces on seagrass under selection pressures of several decades.

### DNA Extraction and Genotyping

Genomic DNA was isolated from leaf tissue using DNeasy plant kits (QIAGEN) following the manufacturer’s instructions. All samples were genotyped using nine polymorphic microsatellite markers previous developed for this species; NSWZos02, NSWZos15, NSWZos18, NSWZos19, NSWZos23, NSWZos25, NSWZos29, NSWZos38, and NSWZos46 (Sherman et al., 2012). Microsatellites were amplified using a polymerase chain reaction (PCR) conducted in 11 μL volumes containing; 10 ng of genomic DNA; 5 μL PCR Master Mix (Qiagen, United States) and 4 μL primer multiplex (0.26 μM of each forward primer and fluorescent dye, 0.13 μM of reverse primer). Thermal cycling conditions for the PCR were; initial hot start at 94°C for 15 min; ten cycles of 94°C for 45 s, 55°C for 45 s, 72°C for 45 s; ten cycles of 94°C for 45 s, 53°C for 45 s, 72°C for 45 s; 20 cycles of 94°C for 45 s, 50°C for 45 s, 72°C for 45 s; final elongation at 72°C for 15 min. PCR amplicons were electrophoresed using an ABI 3130xl Genetic Analyzer, incorporating LIZ 500 (-250) size standard (Applied Biosystems). Alleles were scored using GeneMapper, v3.7 (Applied Biosystems).

### Estimates of Genetic and Genotypic Diversity

We calculated three genetic diversity estimates; allelic richness (AR), expected heterozygosity Nei’s (1978) unbiased estimate H_E_) and observed heterozygosity (H_O_). Estimates of genetic diversity were calculated using only unique multi-locus genotypes as clonal reproduction may influence estimates of population structure. Allelic richness was calculated per locus and population, and standardized based on the smallest sample size at a site (*n* = 13) in the program FSTAT 2.9.3.2 (Goudet, 2002). Observed and expected heterozygosity were calculated using the statistical program GenAlex (Peakall and Smouse, 2012).

Genotypic diversity (*R*) was calculated as the number of unique multi-locus genotypes (*N_G_*) relative to the number of samples collected (*N*) and expressed as: $R=\frac{\left(N_{G}-1\right)}{\left(N-1\right)}$ (Dorken and Eckert, 2001) using the program Genclone (Arnaud-Haond and Belkhir, 2007). Simpson’s diversity index D^∗^ was used to determine clonal heterogeneity (D^∗^ = 1 - Σπ^2^), where π is the frequency of the multi-locus genotype detected in the sample. This index describes the probability of encountering distinct multi-locus genotypes when randomly taking two individuals from the sample. Multi-locus genotypes can occur through resampling of the same clone (asexual reproduction), the recombination of the same alleles in different individuals, somatic mutation or scoring error. The probability of identical multilocus genotypes arising from different sexual reproductive events was assessed by calculating the probability of identity, P_ID_, for each site across all loci (Waits et al., 2001) using the program GenAlex (V6) (Peakall and Smouse, 2012). P_ID_ calculates the probability that two individuals drawn at random within a population will have the same multilocus genotype, and it can be used to estimate the expected number of individuals with the same multilocus genotype within samples. The probability of identity (P_ID_) calculated for each site was low, ranging from P_ID_ = 8 × 10^-5^ at Site 6 to P_ID_ = 9.8 × 10^-7^ at Site 8, indicating the marker system had a high degree of power to identify unique multilocus genotypes.

### Statistical Analyses

We first used regression analysis to test the relationship between genotypic (R) and genetic diversity (AR/He/Ho). We then tested for differences between high and low disturbance sites on each dependent variable separately. We used a hierarchical ANOVA with the main factor Disturbance having two, fixed levels (High and Low). The 2 sites with no disturbance were included in Low disturbance. The random factor, Site, was nested within Disturbance, with 3 quadrats in each Site. Because R varied significantly between levels of disturbance, we also regressed seagrass morphological data against R, for the twelve sites for which morphological measurements were available. This analysis was done in two steps. First, we tested all morphological variables together, using a multivariate linear regression. We then analyzed each individual morphological variable in separate regressions.

## Results

### Levels of Genetic and Genotypic Diversity

We genotyped 630 *Z. muelleri* samples from 14 sites across 11 loci. Overall, we ascertained high levels of genotypic diversity with a total of 397 unique multilocus genotypes detected across all sites. A total of 116 replicate genotypes was identified. The largest clone at any site showed a single genotype sampled 16 times. Numbers of clones per site ranged from 5 to 10. Global genotypic diversity was relatively low (*R* = 0.55), and ranged between 0.30 and 0.82 (**Table 1**). Levels of clonal heterogeneity across sites varied from 0.99 to 0.83.

**Table 1:** Summary of genotypic and genetic diversity measures for *Zostera muelleri* in Moreton Bay.

Site	MLG	R	AR	Ho	He	Fis
1	19	0.41	2.79	0.42	0.39	-0.12
2	14	0.30	2.65	0.43	0.44	-0.03
3	18	0.39	2.88	0.41	0.41	-0.04
4	16	0.34	2.56	0.43	0.44	-0.02
5	33	0.73	3.54	0.46	0.46	-0.03
6	14	0.30	2.21	0.41	0.39	-0.13
7	30	0.66	2.92	0.42	0.41	0.07
8	37	0.82	3.10	0.37	0.41	0.07
9	36	0.80	3.27	0.39	0.40	0.03
10	19	0.41	2.98	0.43	0.43	-0.05
11	33	0.73	3.62	0.39	0.43	0.04
12	26	0.57	3.26	0.45	0.42	-0.06
13	37	0.82	3.14	0.42	0.37	-0.12
14	26	0.60	3.27	0.36	0.37	0.03
Total	397	0.55	3.01	0.42	0.42	-0.03

*MLG, number of multilocus genotypes; R, clonal richness; AR, allelic richness; Ho, observed heterozygosity; He, unbiased expected heterozygosity; F_is_, inbreeding coefficient. Site numbering follows Figure 1 in Maxwell et al. (2014) for sites 1 – 12, and the two additional undisturbed sites are numbered 13 and 14. Number of complete genotypes per quadrat was standardized at 13 and thus 39 per site. N = 45 at each location.*

Levels of genetic diversity were similar across all sites (**Table 1**). Mean number of alleles per locus across all sites was 3.01 (1.50 Standard Error), and ranged between 2.21 (0.44) and 3.62 (0.94). Unbiased expected heterozygosity across all sites was 0.42 (0.03), varying between 0.37 (0.12) and 0.48 (0.10). Observed heterozygosities were similar to expected heterozygosities. Overall observed heterozygosity was 0.42 (0.03), ranging from 0.36 (0.26) to 0.46 (0.10). There was no evidence of inbreeding within any site with F_IS_ values ranging between slight heterozygous excess (-0.13, SE 0.06) and slight deficit (0.07, SE 0.06).

### Comparisons of Genotypic and Genetic Diversity, Disturbance Categories and Morphological Characteristics

**FIGURE 3:**
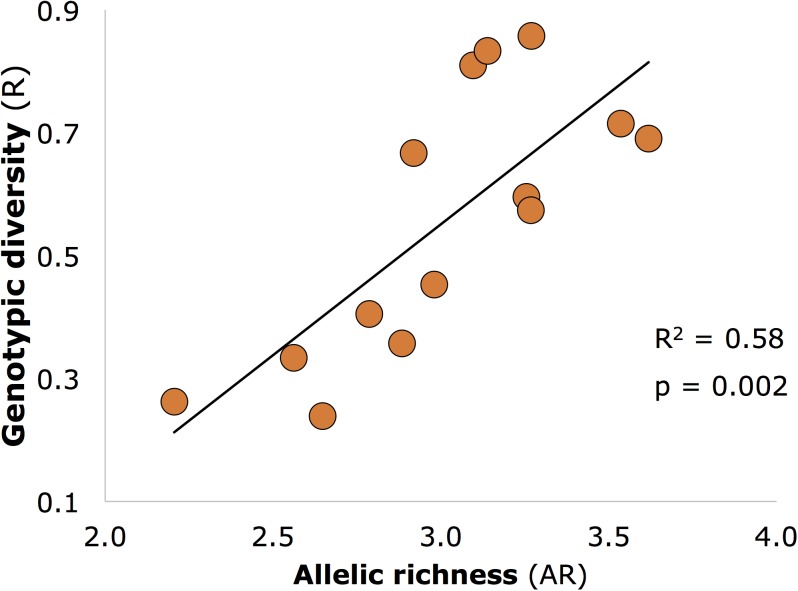
Significant positive relationship between allelic richness (genetic diversity) and genotypic diversity in seagrass meadows of Moreton Bay (*n* = 14 meadows).

Genetic diversity (AR) was significantly positively related to genotypic diversity (**Figure 3**; R^2^ and *p*-values similar for 12 core sites as for all 14 sites shown on figure), but genotypic diversity was not significantly related to expected (He) or observed (Ho) heterozygosity (*p* > 0.05).

**FIGURE 4:**
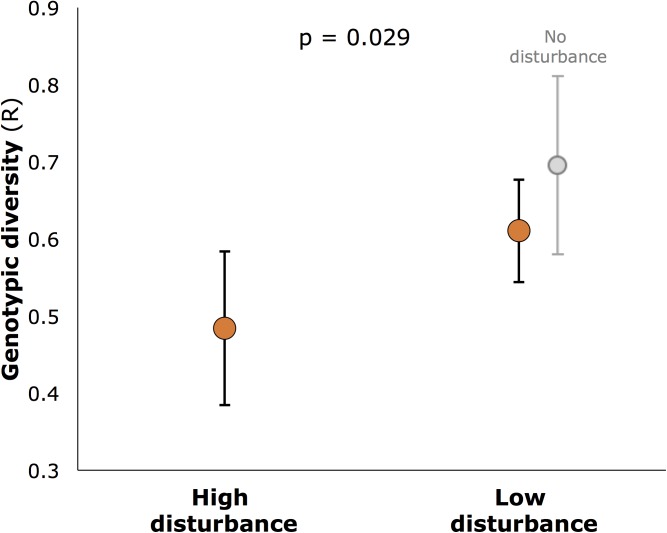
Genotypic diversity (R) in seagrass meadows of Moreton Bay (mean, SE), showing significantly higher diversity in low disturbance meadows (*p*-value is for the main factor, Disturbance, in hierarchical ANOVA (*n* = 6 meadows for each of high and low disturbance; 2 additional meadows with no disturbance shown slightly separated).

Genotypic diversity (*R*) was significantly lower at highly disturbed sites (*R* = 0.49) than at less disturbed sites (*R* = 0.61 for all less disturbed sites, 0.59 for core sites only: **Figure 4**, *p* = 0.029). *R* also varied among sites within disturbance categories (nested factor Site: df 12,28, *F* = 4.413, *p* = 0.001). For genetic diversity, neither AR nor expected heterozygosity (He) differed between disturbed and undisturbed sites (df 1,13, *F* = 0.65, *p* = 0.43 and df 1,13, *F* = 3.68, *p* = 0.065, respectively), but AR varied significantly among sites within disturbance categories (df 12,28, *F* = 2.33, *p* = 0.032).

**FIGURE 5:**
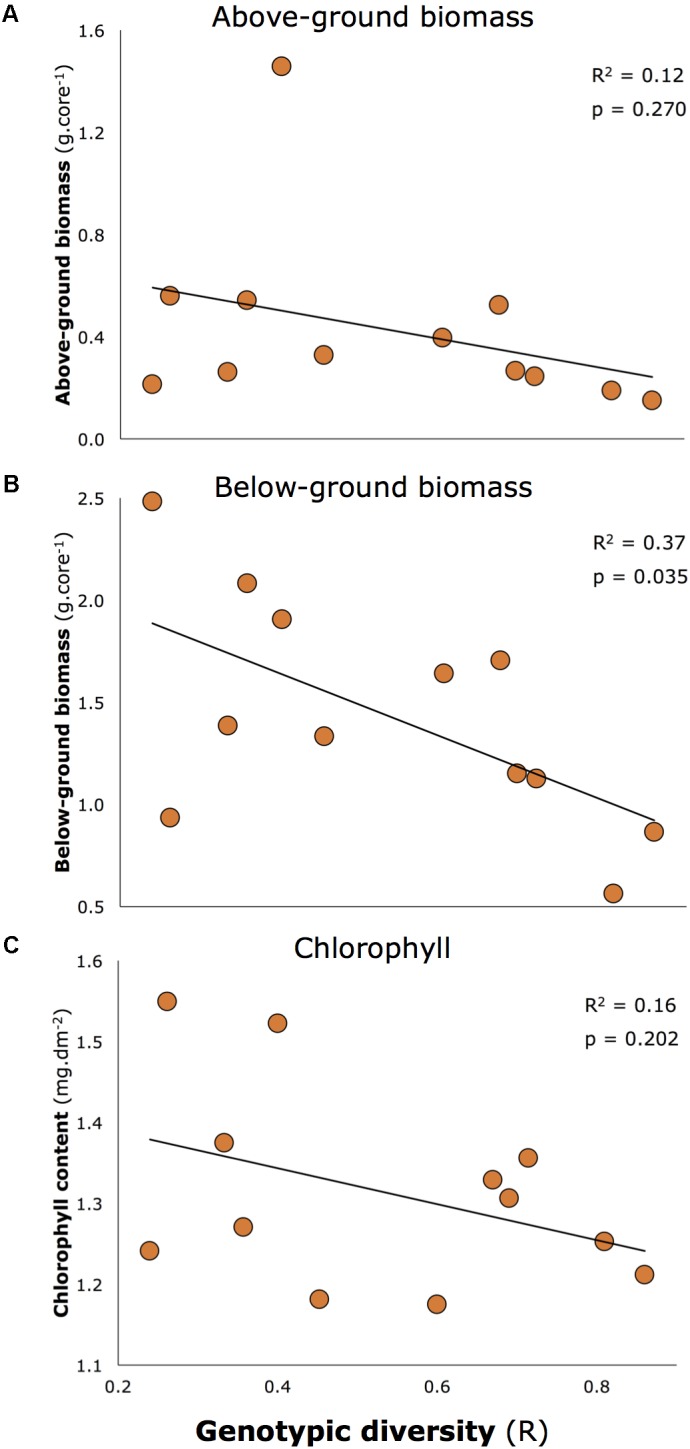
Negative relationships between key morphological characteristics of seagrass and genotypic diversity: **(A)** Above-ground biomass, **(B)** Below-ground biomass, **(C)** Chlorophyll a concentrations. Below-ground biomass is the only significant relationship at 0.05 level, but all relationships are included because of the consistent negative trend.

Genotypic diversity declined with two morphological characteristics reported to be associated with resilience of seagrasses in Moreton Bay in Maxwell et al. (2015) and Maxwell (2014): seagrass biomass (both above and below-ground), and chlorophyll concentrations (**Figure 5**). Overall, these relationships were close to being significant (multivariate linear regression: *p* = 0.053). When tested individually, one was significant at the critical level of 0.05 (all *p*-values shown on **Figure 5**), but the consistent negative relationship is important to note.

## Discussion

The main finding of this study is that in Moreton Bay, seagrass meadows subject to long-term disturbance show greater resilience to a severe perturbation yet have lower genotypic, but not genetic diversity. We interpret this to be a case of highly disturbed sites having selected for a narrow range of genotypes that are able to cope with the harsher conditions. The trend of low genotypic diversity being associated with several morphological fitness parameters supports the conclusion that intense selection in disturbed populations has resulted in more resilient genotypes.

Although a majority of previous studies examining seagrass responses to stressors show a positive trend of higher diversity providing a greater capacity to resist or recover (Jahnke et al., 2015; Salo and Gustafsson, 2016), there are two examples that also show a negative relationship between genotypic diversity and the resilience of seagrass to disturbance (Diaz-Almela et al., 2007; Arnaud-Haond et al., 2010). Lower mortality (i.e., increased resistance) in *P. oceanica* exposed to pollution from fish farms was found to be correlated with lower genotypic diversity (Diaz-Almela et al., 2007). A broader-scale study of 30 meadows of *P. oceanica* subject to a range of different stressors showed the same trend of low genotypic diversity at more resilient sites (Arnaud-Haond et al., 2010). These cases could simply reflect successful competition by a limited number of clones under high disturbance, as we are suggesting for the findings from Moreton Bay. However, in both of the *P. oceanica* studies, the lower genotypic diversity went hand-in-hand with larger clone size (Diaz-Almela et al., 2007), and the presence of widespread, dominant clones potentially provides superior phenotypic plasticity or physiological tolerances and hence an alternative explanation for greater resilience to perturbations (Arnaud-Haond et al., 2010). The possible importance of clone size in *Z. muelleri* in Moreton Bay is a topic worthy of further testing.

Many other studies show a positive relationship between genetic diversity and resilience of seagrasses to disturbances ranging from warming water (Reusch et al., 2005; Ehlers et al., 2008) to grazing and experimental removal of biomass (Hughes and Stachowicz, 2004; Hughes et al., 2010). These studies all used experimental manipulations either in the field or in mesocosms. Such experiments typically are better able to control the relationship between genetic and genotypic diversity, and thus clone size (Arnaud-Haond et al., 2010). In contrast, the three studies with opposing findings - the current study; Diaz-Almela et al. (2007), Arnaud-Haond et al. (2010) – use what Salo and Gustafsson (2016) term natural experiments, measuring genetic diversity in meadows exposed to different levels of uncontrolled disturbances over large scales in field settings.

One limitation of our large-scale natural experiment in Moreton Bay is the lack of information on levels of genotypic diversity prior to disturbance. The greatly increased disturbance from deposition of riverine sediment since European settlement is well cataloged (Leigh et al., 2013), but without the pre-disturbance genetic data it is difficult to make strong inferences about how original levels of genotypic diversity have changed, and how they determined resilience (Arnaud-Haond et al., 2010; Hughes, 2010). Looking forward, one important consideration is that while the intense disturbance history on some seagrass meadows in Moreton Bay may have resulted in locally adapted populations, the loss of genotypic diversity from these sites may constrain their future adaptive potential (Antonovics, 1971; Linhart and Grant, 1996; Tiffin and Ross-Ibarra, 2014). This possibility comes with a major caveat, however, that at this stage adaptive capacity resulting from sexual reproduction in Moreton Bay is unknown. There are no records of seed production rates or dispersal of *Z. muelleri* or any other seagrass species in Moreton Bay. The only study of dispersal in *Z. muelleri* from a resilience perspective is a test of recovery in small patches, 300 mm in diameter, from which seagrass had been removed; no recovery from sexual reproduction occurred over the year-long experiment (Macreadie et al., 2014b). Sexual reproduction was demonstrably less important than asexual reproduction, although care should be taken in applying conclusions from the experiment by Macreadie et al. (2014b) in the temperate waters of an estuarine lake to the subtropical waters of Moreton Bay. At this stage, the role of sexual reproduction in adaptive capacity of *Z. muelleri* in Moreton Bay remains an open question.

Our measure of genetic diversity, as for previous studies of genetic diversity and disturbance in seagrass, uses a small number of neutral markers. This technique cannot be expected to capture the full adaptive potential of plants (Macreadie et al., 2014b; Whitlock, 2014). With the recent publication of a draft genome for *Z. muelleri* (Lee et al., 2016) and the development of more affordable next generation sequencing approaches for non-model taxa (da Fonseca et al., 2016; Sherman et al., 2016), it is now possible to genotype individuals across a large number of single nucleotide polymorphism (SNP) markers. Identification of SNPs with signatures of selection, i.e., either directly under selection or close to regions under selection, should in future make it possible to assess the importance of genetic diversity on adaptability of populations of clonal organisms and better understand the role of genetic versus genotypic diversity for resilience (Savolainen et al., 2013).

The responses of seagrass in Moreton Bay have provided an informative test of the role of genotypic diversity. Our interpretation of how genetic structure is affected by disturbance benefitted substantially from past studies of morphometric and physiological responses of seagrass to stressors in the bay. In terms of understanding the mechanisms through which genotypic diversity influences resilience (Hughes et al., 2008), Moreton Bay is now well placed for using transplant experiments to test for local adaptation (Gibson et al., 2016; York et al., 2017), and mesocosm experiments to test the range of physiological tolerances of genotypes from highly impacted versus less impacted sites.

## Author Contributions

RC, TS, PSM, AO, PIM, and CS conceived, conceptualized, and interpreted the project, and revised and edited the manuscript. PSM and AO collected the field data. TS and CS ran the laboratory analyses. RC, CS, TS, and PSM performed the statistical analyses. RC and CS wrote the manuscript. RC and CS acquired funding.

## Conflict of Interest Statement

The authors declare that the research was conducted in the absence of any commercial or financial relationships that could be construed as a potential conflict of interest. The handling Editor is currently co-organizing a Research Topic with one of the authors PIM, and confirms the absence of any other collaboration.
