# Supplementary material for: Highly Disturbed Populations of Seagrass Show Increased Resilience but Lower Genotypic Diversity
Source: Front Plant Sci. 2018 Jun 29;9:894. doi: 10.3389/fpls.2018.00894 (PMC6034141; doi:10.3389/fpls.2018.00894)
Supplement: Supplementary file 1 [file Data_Sheet_1.DOCX]

**Appendix 1**

**Description of methods for multivariate assessment of feedbacks indicating resilience in seagrass** (from Maxwell 2014)

We measured three feedback processes that are critically important to the persistence of seagrass meadows in eutrophic and turbid waters: rate of grazing of epiphytic algae, suppression of sediment resuspension, and nutrient uptake from the water column. The rates measured for these three processes were then analysed in a multivariate Canonical Analysis of Principal Coordinates (CAP), providing a single quantitative measure ranging between +1 (very high rates) and – 1 (very low rates).

Potential grazing pressure on seagrass epiphytes was quantified by measuring consumption of red algae, *Catenella nipae*, growing attached to local mangrove pneumatophores. Twelve pneumatophores with epiphytic algae were deployed at each site. The grazing measure was percentage weight loss of algae over a 72 hour deployment, calculated after subtracting the final cleaned weight of the pneumatophore. To check that algal weight loss was due to grazing rather than extraneous factors such as mechanical abrasion against seagrass leaves, one pneumatophore at each site was caged to exclude grazing animals. None of the caged units showed any weight loss during the deployment period, and thus we conclude that weight loss was an effective proxy for grazing pressure.

Sediment resuspension was estimated at each site using light attenuation measured at two heights above the sea floor at each site. Light attenuation was measured using photosynthetic irradiance (PAR) loggers, at 0.2 m, the approximate height of the highest seagrass canopy, and 0.8 m, which is below but nearer to the surface of the water. Light was recorded at ten-minute intervals during the middle of the day (2 hrs either side of solar noon), for seven days. Resuspension of sediment lowers irradiance at the bottom logger, below that expected based solely on water depth. The degree of suppression of resuspension is evident as higher irradiance at the bottom logger, relative to that at the higher logger. Light attenuation (K_d_) in m^-1^ was calculated using the formula: K_d_ = Ln (PAR at depth 1) – Ln (PAR at depth 2) / difference in depth. Smaller negative values result from greater suppression of resuspension.

The processing of nutrients, or nutrient uptake, was measured in three transparent Perspex domes (diam. 29.5 cm, height 20 cm above the sediment interface) deployed at each site for 5 hr starting at 8:30 am. Water samples for nutrient analyses were collected at the start and the end of the incubations. Water samples were analysed for species of dissolved nitrogen concentrations.

**Table Appendix 1. Summary statistics for the rates of three key feedback processes used to calculate the multivariate index shown in Fig. 2 A.**

| **Variable** | **Units** | **High Disturbance** | **Low Disturbance** |
| --- | --- | --- | --- |
| Grazing rate | % removed in 3 days | 20.8 | 11.1 |
| Light attenuation | m^-1^ | -1.25 | -1.50 |
| Nutrient uptake | mg.L^-1^ | 0.006 | 0.001 |
